# Effect of Intravenous Contrast on CT Body Composition Measurements in Patients with Intraductal Papillary Mucinous Neoplasm

**DOI:** 10.3390/diagnostics14222593

**Published:** 2024-11-18

**Authors:** Ranjit S. Chima, Tetiana Glushko, Margaret A. Park, Pamela Hodul, Evan W. Davis, Katelyn Martin, Aliya Qayyum, Jennifer B. Permuth, Daniel Jeong

**Affiliations:** 1 Department of Diagnostic Imaging and Interventional Radiology, H. Lee Moffitt Cancer Center & Research Institute, 12902 USF Magnolia Drive, Tampa, FL 33612, USAdaniel.jeong@moffitt.org (D.J.); 2 Department of Gastrointestinal Oncology, H. Lee Moffitt Cancer Center & Research Institute, 12902 USF Magnolia Drive, Tampa, FL 33612, USA; 3 Department of Biostatistics and Bioinformatics, H. Lee Moffitt Cancer Center & Research Institute, 12902 USF Magnolia Drive, Tampa, FL 33612, USA; 4 Department of Cancer Epidemiology, H. Lee Moffitt Cancer Center & Research Institute, 12902 USF Magnolia Drive, Tampa, FL 33612, USA; 5 Department of Clinical Science, H. Lee Moffitt Cancer Center & Research Institute 12902 USF Magnolia Drive, Tampa, FL 33612, USA

**Keywords:** body composition, skeletal muscle index, intraductal papillary mucinous neoplasm, pancreatic, CT, deep learning

## Abstract

Background: The effect of differing post-contrast phases on CT body composition measurements is not yet known. Methods: A fully automated AI-based body composition analysis using DAFS was performed on a retrospective cohort of 278 subjects undergoing pre-treatment triple-phase CT for pancreatic intraductal papillary mucinous neoplasm. The CT contrast phases included noncontrast (NON), arterial (ART), and venous (VEN) phases. The software selected a single axial CT image at mid-L3 on each phase for body compartment segmentation. The areas (cm^2^) were calculated for skeletal muscle (SM), intermuscular adipose tissue (IMAT), visceral adipose tissue (VAT), and subcutaneous adipose tissue (SAT). The mean Hounsfield units of skeletal muscle (SMHU) within the segmented regions were calculated. Bland–Altman and Chi Square analyses were performed. Results: SM-NON had a lower percentage of bias [LOA] than SM-ART, −0.7 [−7.6, 6.2], and SM-VEN, −0.3 [−7.6, 7.0]; VAT-NON had a higher percentage of bias than ART, 3.4 [−18.2, 25.0], and VEN, 5.8 [−15.0, 26.6]; and this value was lower for SAT-NON than ART, −0.4 [−14.9, 14.2], and VEN, −0.5 [−14.3, 13.4]; and higher for IMAT-NON than ART, 5.9 [−17.9, 29.7], and VEN, 9.5 [−17.0, 36.1]. The bias in SMHU NON [LOA] was lower than that in ART, −3.8 HU [−9.8, 2.1], and VEN, −7.8 HU [−14.8, −0.8]. Conclusions: IV contrast affects the voxel HU of fat and muscle, impacting CT analysis of body composition. We noted a relatively smaller bias in the SM, VAT, and SAT areas across the contrast phases. However, SMHU and IMAT experienced larger bias. During threshold risk stratification for CT-based measurements of SMHU and IMAT, the IV contrast phase should be taken into consideration.

## 1. Introduction

There is growing interest in computed tomography (CT)-based body composition analysis, with artificial intelligence (AI) algorithms now allowing for automated compartment segmentation and quantitative analyses [1]. A vast amount of data is routinely acquired in standard-of-care abdominopelvic CT imaging, which can be used to perform body composition assessments, which have been termed “opportunistic”. The established CT-based body composition metrics currently include measurements of the area of the skeletal muscle, visceral adipose tissue, subcutaneous adipose tissue, and intermuscular adipose tissue [2,3,4]. The easy availability and present underutilization of these data have led to interest in their clinical applications to analyzing body composition, as well as the emergence of new measurement algorithms [5].

While opportunistic body composition data have clear value for screening purposes, a growing body of literature is investigating the predictive value of these data across ranging contexts, from perioperative complications to cancer survival. For example, body composition metrics have shown promise for predicting perioperative course. Visceral obesity has been implicated as a predictor of abdominal wall dehiscence, while decreased muscle bulk and myosteatosis have been linked to increased perioperative mortality in the critically ill [6,7].

An increasing body of work shows the promising prognostic value of body composition data in multiple different cancers. Still, the heterogeneity in CT body composition analyses presents a challenge for the meaningful application of these techniques. In a meta-analysis and systematic review, Cheng et al. compared the influence of visceral, subcutaneous, and total adipose tissue on cancer survival metrics for breast, colorectal, gastroesophageal, head and neck, hepatocellular, ovarian, pancreatic, prostate, and renal cell cancer cohorts. While the authors were not able to establish an association between body composition measurements and survival, they did note significant heterogeneity in the measurement techniques across different study designs [8]. Yet more focused studies performed in specific cancer populations (esophageal, gastric, lung, and colorectal) have found associations between adiposity and skeletal muscle measurements with prognostic factors [9,10,11,12,13]. This suggests that a better standardization of body composition techniques could allow for more comprehensive cross-study analysis. Beyond survival, body composition data showed predictive value for general markers of wellness in the oncologic patient population, such as lean body mass, walking distance, and sarcopenia, in studies by Yip et al. and Brown et al. [14,15].

The predictive performance of body composition metrics for chronic pancreatic disease and pancreatic cancer patients is a continuing topic of exploration [8]. A 2019 prospective study by Bieliuinene et al. found utility in body composition metrics for the detection of sarcopenia and osteopenia in a group of 100 patients with chronic pancreatitis and pancreatic ductal adenocarcinoma [16]. Several studies have demonstrated that sarcopenia and visceral obesity have predictive value for perioperative complication rate and severity following pancreaticoduodenectomy [17,18,19]. Furthermore, body composition measures have also shown predictive value for mortality in pancreatic cancer [20,21]. Prior authors also showed that presurgical CT-based visceral adiposity may play a role in predicting the pathology of malignant intraductal papillary mucinous neoplasm (IPMN) [22]. While this paper is focused on the different impacts of contrast phases on body composition measurements, the utility of these measurements for intraductal papillary mucinous neoplasm (IPMN) remains a topic of further investigation.

Validated landmark studies by Shen et al. and Mourtzakis et al. found that noncontrast axial CT imaging at the mid-L3 vertebral body level is representative of total body composition [3,4]. Early methods of body composition relied on manual body compartment segmentation and per-voxel Hounsfield unit (HU) thresholds for classifying fat or muscle, known as the Alberta Protocol [4,23]. Notably, the earliest studies were performed on noncontrast CT which did not account for the influence of intravenous contrast. Advancements have led to the development of multiple semi- and fully automated body composition measurement software solutions using artificial intelligence [2,24]. CT has advantages over other modalities, including Magnetic Resonance Imaging (MRI), due to the discrete and absolute Hounsfield units assigned to each imaged voxel in CT, which mitigates the need for voxel-based pre-normalization. Most MRI (non-mapped) voxel signal values are relative values and benefit from data resampling or normalization prior to comparing the signal values within a cohort, which can be resource-intensive. Additionally, Dual-Energy X-Ray Absorptiometry (DEXA) also offers accurate body composition analysis [25]. However, the output images in DEXA are 2D, with a limited ability to evaluate focused regions of the body, versus CT allowing for axial slice-by-slice or volumetric analyses based on the coverage field of view [26].

One challenge that has arisen in this field is that many standard-of-care cohorts in clinical practice routinely use contrast-enhanced CT imaging. These exams may vary considerably in the contrast phase timing depending on the institutional protocol and individual factors. Furthermore, normative values are not yet well established regarding how IV contrast affects the HU measurements of various tissue types and how this might impact body composition measurements in a standard cancer patient population. Adipose and muscular tissues contain varying degrees of vascularity, which could then undergo enhancement during IV contrast injection and alter the HU of specific voxels. In differentiating muscle from fat in CT body composition analysis, IV contrast could influence the HU of CT voxels that are near these cutoff levels, therefore affecting the total areas or volumes of fat versus muscle. While multiple studies have explored how portal venous phase imaging affects body composition measurements, there is a need for additional data from multiphasic exams [27,28]. Therefore, the effect of IV contrast at different phases is crucial information to allow for more widespread application of body composition assessments.

The goal of this study is to explore whether intravenous contrast may bias different body composition metrics when they are obtained at pre- and differing post-contrast phases. This understanding will permit wider applicability of body composition analysis to contrast-enhanced CT exams, as well as putting the thresholds previously established for these metrics into context.

## 2. Materials and Methods

A retrospective cohort of 278 subjects undergoing pre-treatment triple-phase CT for intraductal papillary mucinous neoplasm (IPMN) was selected for body composition analysis. This was an opportunistic cohort, as IPMN patients routinely undergo this preoperative imaging at our institution (an NCI-designated cancer center). The CT parameters and settings are detailed below in Table 1.

A topogram was taken in the AP view only. Pre-monitor scanning was performed at the level of the celiac axis. A cup of water was administered by mouth to the patient 30 min prior to the scan. Iopamidol 76% IV contrast (Bracco Diagnostics, Monroe Township, NJ, USA) was administered as the IV contrast agent for CT. The volume administered was weight-based, with a 100 mL minimum at the following rates based on weight: at 3.5 mL/s. (≥60 kg) or 3.0 mL/s. (<60 kg)

**Figure 1:**
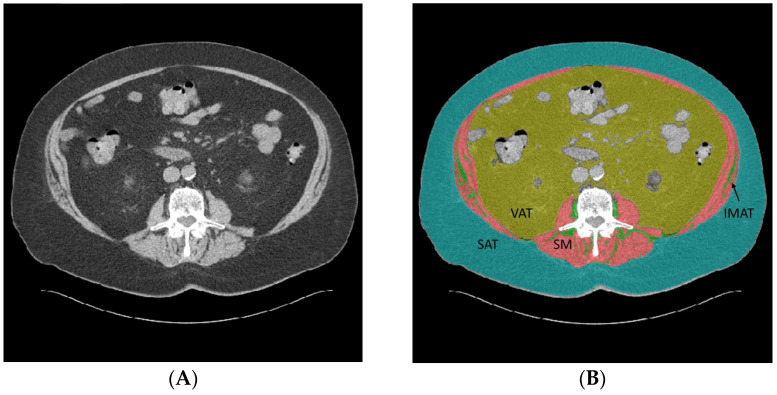

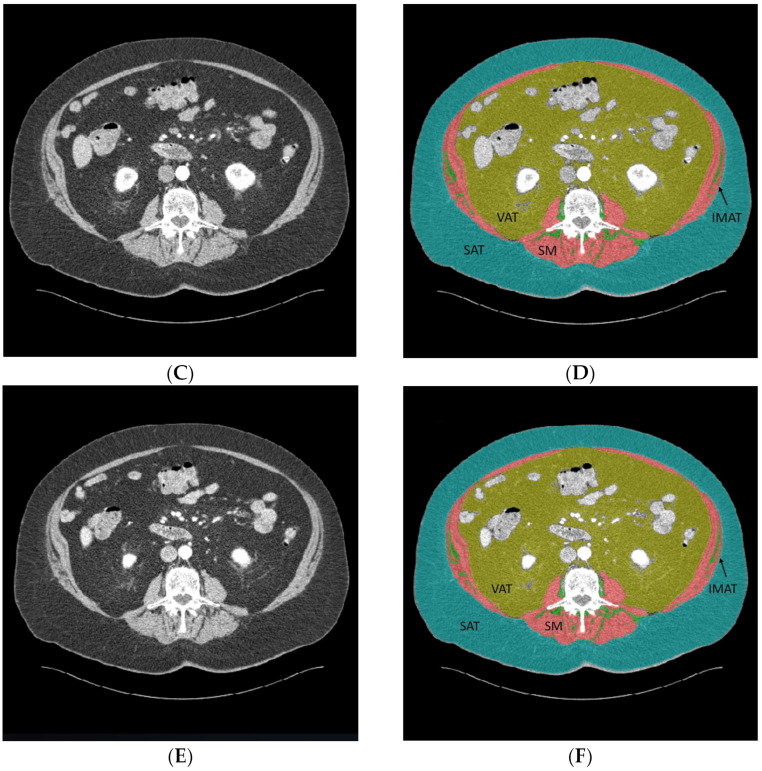
(**A**) Noncontrast CT and (**B**) DAFS v3 segmented noncontrast CT at the axial mid-L3 level. In each segmented image, red segmentation represents the skeletal muscle (SM) area. Teal segmentation represents subcutaneous adipose tissue (SAT). Olive green represents visceral adipose tissue (VAT). Bright green represents intermuscular adipose tissue (IMAT; corresponds to tissue designated by the black arrows among skeletal muscle). (**C**) Arterial phase post-contrast CT and (**D**) segmented arterial CT at the same level. (**E**) Venous phase post-contrast CT and (**F**) segmented venous CT at the same level.

Body composition segmentation and quantification were performed using the DAFS v3 software (Voronoi Health Analytics; Vancouver, BC, Canada), which has previously been validated for body composition measurements [29]. During processing, the software selected an axial CT image at the mid-L3 vertebral body level in noncontrast (NON), arterial (ART), and portal venous (VEN) phase imaging, and automated segmentations delineating the body composition compartments were performed, as seen in Figure 1. DAFS software segmented and calculated the skeletal muscle area cm^2^ (SM), the visceral adipose tissue area cm^2^ (VAT), the subcutaneous adipose tissue area cm^2^ (SAT), and the intermuscular adipose tissue area cm^2^ (IMAT). The mean Hounsfield units within the segmented skeletal muscle were also calculated (SMHU). SM was indexed to height squared to calculate the skeletal muscle index (SMI), which is a widely reported biomarker for sarcopenia [30]. Output images from the DAFS software were reviewed by a radiologist to assess clinically significant artifacts and the segmentation quality.

Subjects were categorized as viscerally obese at each contrast phase based on the prior suggested threshold of VAT > 100 cm^2^ [30]. Additionally, subjects were categorized at each contrast phase as sarcopenic based on the prior reported thresholds of an SMI < 46.96 cm^2^/m^2^ in males and an SMI < 32.46 cm^2^/m^2^ in females [31].

Bland–Altman analysis of the body composition values was performed between noncontrast–arterial measurements and noncontrast–venous measurements for each metric. The bias and limits of agreement (LOA) were provided in absolute and relative percentage values. Medcalc (Medcalc Software version 22, Ostend, Belgium) was used to perform the Bland–Altman analyses and generate plots. Conventional quantitative and qualitative clinical data were also compared between the groups, via Chi Square (categorical), using SPSS version 26 (IBM Corp., Armonk, NY, USA) to assess the agreement of each contrast in defining the body composition parameters.

## 3. Results

In 278 subjects (140 females; age: 69.2 ± 11.0 years) with pathologically proven IPMN undergoing pre-therapy triple-phase contrast-enhanced CT exams, their average HU of skeletal muscle in the ART (*p* < 0.01) and VEN (*p* < 0.01) phases were higher than SM NON. The average IMAT-VEN (*p* = 0.01) was lower than IMAT-NON.

Patients with low-quality CT images were excluded from this analysis. During the quality review of the CT imaging by the radiologist, a single case had a severe artifact related to spinal hardware, and this case was excluded from all the analyses. Bland–Altman analysis in absolute cm^2^ showed that SM-NON had lower bias [LOA] than SM-ART −0.6 [−7.2,5.9]. SM-NON had a lower bias than SM-VEN, −0.3 [−7.4,6.9]; VAT-NON had a higher bias than VAT-ART, 3.2 [−20.5,26.9]. VAT-NON had a higher bias than VAT-VEN, 5.3 [−18.1,28.7]; SAT-NON had a lower bias than SAT-ART, −0.8 [−21.4,19.8]. SAT-NON had a lower bias than SAT-VEN, −1.3 [−26.5,23.9]; IMAT-NON had a higher bias than IMAT-ART, 1.0 [−3.4,5.3]. IMAT-NON had a higher bias than IMAT-VEN, 1.5 [−3.6,6.6]. The bias in SMHU NON [LOA] was lower than that in both SMHU-ART, −3.8 HU [−9.8,2.1], and SMHU-VEN, −7.8 HU [−14.8,−0.8].

**Figure 2:**
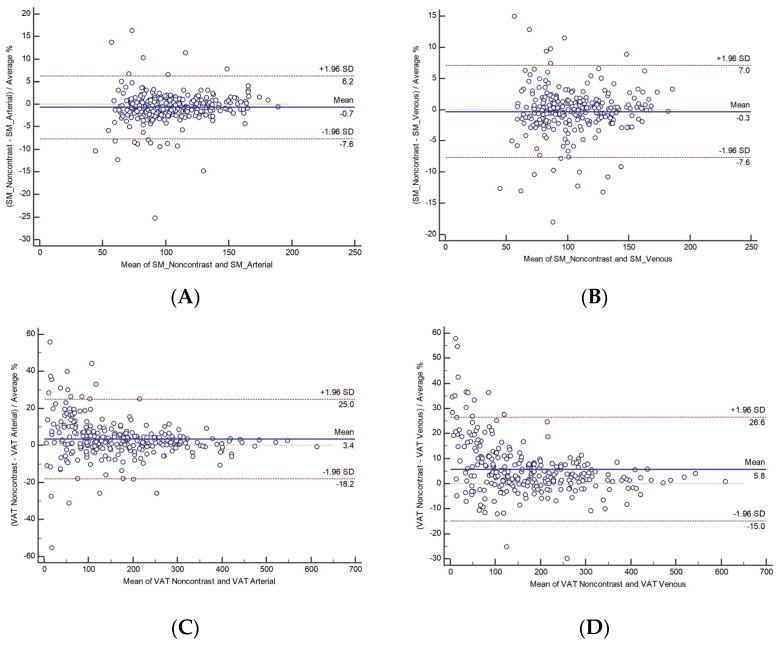

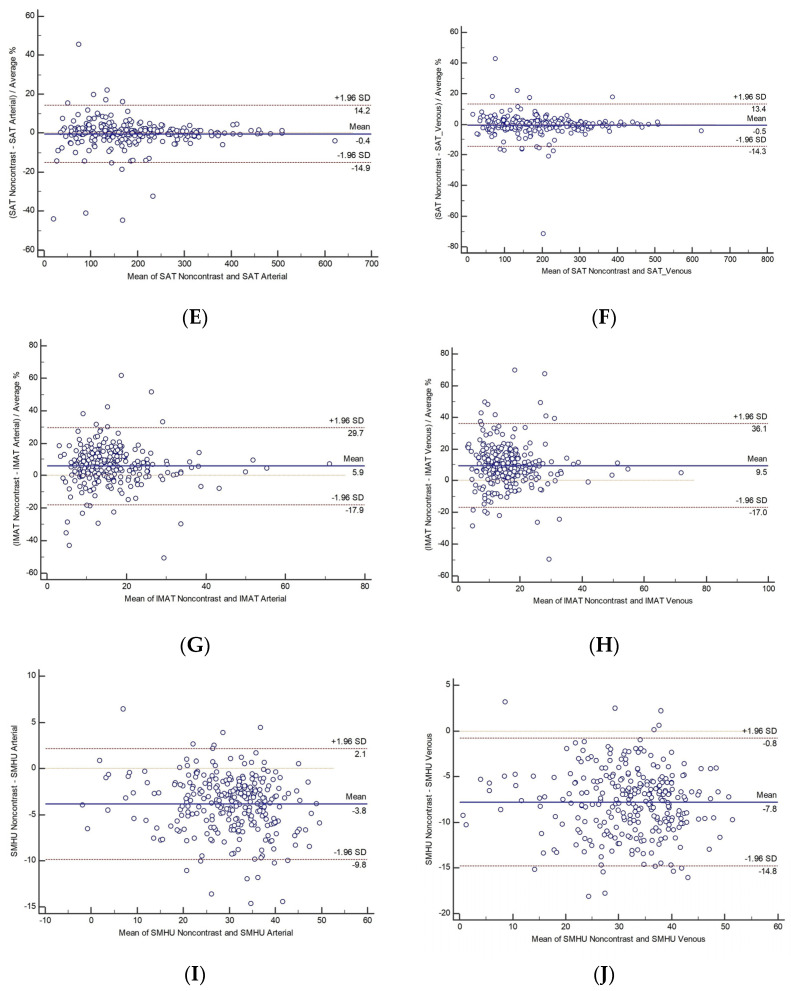
Bland–Altman plots: noncontrast phase skeletal muscle area (SM) compared to (**A**) arterial phase SM and (**B**) venous phase SM. Noncontrast phase visceral adipose tissue area (VAT) compared to (**C**) arterial phase VAT and (**D**) venous phase VAT. Noncontrast phase subcutaneous adipose tissue area (SAT) compared to (**E**) arterial phase SAT and (**F**) venous phase SAT. Noncontrast phase intermuscular adipose tissue area (IMAT) compared to (**G**) arterial phase IMAT and (**H**) venous phase IMAT. Noncontrast phase Hounsfield units of skeletal muscle (SMHU) compared to (**I**) arterial phase SMHU and (**J**) venous phase SMHU. The horizontal lines represent reference lines with respect to the data distribution drawn at 1.96 standard deviations below the mean (lower line), the mean (middle line), and 1.96 standard deviations above the mean (top line).

The Bland–Altman percentage analysis (Figure 2) showed that SM-NON had a lower %bias [LOA] than SM-ART, −0.7% [−7.6,6.2] (Figure 2A), and SM-VEN, −0.3% [−7.6,7.0] (Figure 2B); VAT-NON had a higher percentage of bias than ART, 1.9% [−11.9,15.6] (Figure 2C) and VEN, 3.1% [−10.6,16.7] (Figure 2D); SAT-NON had a lower percentage of bias than ART, −0.4% [−11.2,10.4] (Figure 2E), and VEN, −0.6% [−13.8,12.6] (Figure 2F); IMAT-NON had a higher percentage of bias than ART, 5.9% [−20.6,32.3] (Figure 2G), and VEN, 9.2% [−22.3,40.8] (Figure 2H); and SMHU NON had a lower percentage of bias than ART, −9.3% [−128.0,109.3] (Figure 2I), and VEN, −34.4% [−215.1,−0.8] (Figure 2J). These results are summarized in Table 2.

The contrast-phase-based stratification based on the prior thresholds for visceral obesity is provided in Table 3. In general, IV contrast was attributed to a decrease in visceral fat, and subsequently, fewer subjects qualified as viscerally obese at the arterial (*p* = 0.24) and venous (*p* = 0.36) phases compared to the noncontrast phase, respectively. Likewise, IV contrast resulted in fewer subjects surpassing the sarcopenia threshold at the arterial (*p* = 0.48) and venous phases (*p* = 0.79) compared to the noncontrast phase, respectively. During the quality review of the CT imaging by the radiologist, a single case had a severe artifact related to spinal hardware, and this case was excluded from the analysis.

## 4. Discussion

This study evaluated standard CT-based body composition metrics at multiple contrast phases in patients with IPMN to assess for the agreement and bias. Our results show that intravenous contrast most significantly affects measurements of the area of intermuscular adipose tissue (IMAT) and voxel Hounsfield units of muscle, which can therefore affect body composition analyses. Conversely, a relatively small bias was demonstrated between the contrast phases in the skeletal muscle (SM), visceral adipose (VAT), and subcutaneous adipose tissue (SAT) measurements.

IMAT represents the relatively thicker strands of adipose tissue located within segmented regions of muscle and has been suggested as a modulator of insulin sensitivity [32]. IMAT is also believed to be involved in triggering local and systemic low-grade inflammatory responses [32]. On post-contrast CT imaging, branching and perforating arteries and veins are often visualized within the IMAT regions. The marked increase in HU observed during contrast injection causes the vessels and perivascular voxels to increase in HU, partly in relation to volume-averaging. Since quantification of IMAT relies on the voxel HU representing the fat values, as determined by the DAFS software’s algorithms, this increase could cause “fat” voxels on noncontrast scans to include HU that are “non-fat” or even represent muscle density in post-contrast imaging. This could explain the significant decrease in the area of IMAT we report at both the ART and VEN phases compared to the NON phase.

Likewise, the increased density of post-contrast skeletal muscle perforating the arteries and veins within the muscle could contribute to the increased density of the voxels of muscle tissue. The HU of skeletal muscle would subsequently increase relative to the noncontrast phase value. This would explain the increased SMHU we reported between the noncontrast phase and both the arterial and venous phases.

When comparing the contrast phases using Bland–Altman analysis, the absolute and percentage biases were used to demonstrate the relative differences in the body composition areas across the cohort. For patients with relatively small areas for fat or muscle, smaller absolute differences between the contrast phases yielded larger percent differences. Similarly, small absolute differences in the HU of muscle between phases can result in very large percentage changes when the average HU are very close to zero HU. However, the percentage-based Bland–Altman analysis helped to demonstrate the relative changes in the areas of VAT, SAT, and IMAT observed between contrast phases.

The clinical application of body composition measurements often focuses on risk-stratifying a patient with sarcopenia or visceral obesity. Visceral obesity has previously been defined as VAT ≥ 100 cm^2^ for males and females at the third lumbar vertebral axial level [31]. Sarcopenia has previously been defined as having a skeletal muscle index of <46.96 cm^2^/m^2^ for males or <32.46 cm^2^/m^2^ for females [30]. Table 3 shows the arterial and venous post-contrast phases led to fewer subjects being classified as viscerally obese compared to the noncontrast phase; however, these were non-significant differences. Similarly, the arterial and venous post-contrast phases led to fewer subjects being classified as sarcopenic, although these were also non-significant differences, with the agreement between the noncontrast and post-contrast phases representing a high percentage (≥93.3%). Overall, the presence of IV contrast may have contributed to the perivascular fat voxels at the noncontrast phase having increased HU and falling into the skeletal muscle range in post-contrast imaging in a small number of cases.

While other studies have explored the effect of the IV contrast phase on body composition metrics, these studies used locally developed and refined algorithms [28,33]. Our study tested the widely commercially available DAFS v3 platform, which currently may have wider availability. Perez et al. focused on pre-contrast versus post-contrast portal venous phase imaging (excluding the arterial phase, related to field-of-view coverage), while our study also included the arterial phase in its analysis [33].

Our study had several limitations. During the three-phase CT exam, each patient underwent three CT scan passes, with identical breath hold instructions during the exam. Slight differences in breath hold between the contrast phases could theoretically alter the specific fat and muscle located at the mid-L3 axial level, particularly for larger patients. Additionally, bowel peristalsis could occur within the short intervals between the contrast phases, which could displace the precise location of the visceral fat versus the bowel, which lines up with the L3 axial level. Representative subtle differences in the anatomic structures in a mid-L3 axial image can be appreciated in Figure 1. We observed larger absolute and relative differences in the measurement of VAT compared to those of SAT, likely related to the potential variability in the position of L3 visceral fat between phases. SAT would be expected to be less variable due to the lack of organs and other structures located in the SAT region. IMAT is the smallest absolute area that we measured, and relatively small absolute changes in IMAT can lead to large percentage-based changes which may be clinically relevant.

Future studies that evaluate visceral fat in 3D and volumetrically would be less affected by subtle differences in breath holding and bowel peristalsis. Our cohort of IPMN patients was an opportunistic group who had systematic access to multiphasic post-contrast CT, which is not routinely available for many oncologic cohorts or healthy volunteer groups. The majority of IPMN patients will also undergo abdominal MRI/MRCP for initial assessment and surveillance. Although the presence of cancerous or precancerous pancreatic lesions can affect the absolute body composition of any subject, our study only compared the body composition metrics within the same patient and the same CT exam across contrast phases to isolate the effect of the contrast phases on body composition.

## 5. Conclusions

The results show that intravenous contrast can cause bias in quantifying the areas and Hounsfield units of muscle and intermuscular adipose tissue, which can therefore affect body composition analyses. The largest biases reported in our study found the venous post-contrast phase increased the HU of skeletal muscle by 7.8 HU and the venous post-contrast phase decreased the area of IMAT by 9.2%. When referencing the previously established threshold values for normal and abnormal SMHU and areas of IMAT, specific attention to the IV contrast phases would help to ensure identical contrast phases are being compared. Future studies could further explore the bias from IV contrast phase on 3D volumetric body composition measurements compared to the 2D body composition measurements reported in this study.

## Figures and Tables

**Table 1:** Triple-phase CT protocol for preoperative assessment of IPMN patients at our institution. Exams performed using Siemens scanners.

Acquisition Parameter	Noncontrast (NON)	Arterial Phase (ART)	Venous Phase (VEN)
Type	Helical	Helical	Helical
kVp	120	120	120
mA	Care dose	Care dose	Care dose
CARE Dose4D (Quality ref mAs)	On (150)	On (150)	On (150)
Rotation time (s)	0.5	0.5	0.5
SFOV/DFOV (cm)	50 cm/patient size	50 cm/patient size	50 cm/patient size
Slice thickness (mm)	3.0	3.0	3.0
Direction	Craniocaudal	Craniocaudal	Craniocaudal
Breathing instructions	Held inspiration	Held inspiration	Held inspiration
Scan delay	None	ROI-triggered: abdominal aorta near the celiac axis at 120 Hounsfield units	60 s post-injection
Coverage	Diaphragm to iliac crest	Diaphragm to iliac crest	Diaphragm to iliac crest
Reconstruction thickness/increment (mm)	3.0 × 3.0	3.0 × 3.0	3.0 × 3.0
Kernel/window	I30F/abdomen	I30F/abdomen	I30F/abdomen

IPMN: intraductal papillary mucinous neoplasm.

**Table 2:** Bland–Altman analyses comparing the noncontrast phase to the arterial and venous phases, respectively, for each of the body composition metrics.

	Noncontrast vs. Arterial Bias [LOA]	Noncontrast vs. Arterial Bias% [LOA]	Noncontrast vs. Venous Bias [LOA]	Noncontrast vs. Venous Bias% [LOA]
Skeletal Muscle (SM)	−0.6 [−7.2,5.9]	−0.7% [−7.6,6.2]	−0.3 [−7.4,6.9]	−0.3% [−7.6,7.0]
Visceral Adipose Tissue (VAT)	3.2 [−20.5,26.9]	1.9% [−11.9,15.6]	5.3 [−18.1,28.7]	3.1% [−10.6,16.7]
Subcutaneous Adipose Tissue (SAT)	−0.8 [−21.4,19.8]	−0.8% [−21.4,19.8]	−1.3 [−26.5,23.9]	−1.3% [−26.5,23.9]
Intermuscular Adipose Tissue (IMAT)	1.0 [−3.4,5.3]	5.9% [−20.6,32.3]	1.5 [−3.6,6.6]	9.2% [−22.3,40.8]
Skeletal Muscle Hounsfield Units	−3.8 HU [−9.8,2.1]	−9.3% [−128.0,109.3]	−7.8 HU [−14.8,−0.8]	−34.4% [−215.1,−0.8]

LOA: limit of agreement.

**Table 3:** Visceral obesity and sarcopenia stratification based on prior CT L3-image-based thresholds [28,29]. The Chi-Square test was used to compare arterial and venous phase outcomes to the noncontrast phase outcome, respectively, with *p*-values provided. *p* < 0.05 was considered statistically significant in this analysis, and none of the differences in outcome between the contrast phases reached statistical significance.

	Noncontrast *n*/We278	Arterial *n*/278	Venous *n*/278
Visceral Obesity VAT ≥ 100 cm^2^	195	182 *p* = 0.24	185 *p* = 0.36
Sarcopenia Male SMI < 46.96 cm^2^/m^2^ OR Female SMI < 32.46 cm^2^/m^2^	182	174 *p* = 0.48	179 *p* = 0.79

## Data Availability

The data presented in this study are available on request from the corresponding author. The data are not publicly available due to the database complexity and local storage guidelines.
